# High Frequency Repetitive Transcranial Magnetic Stimulation Alleviates Cognitive Impairment and Modulates Hippocampal Synaptic Structural Plasticity in Aged Mice

**DOI:** 10.3389/fnagi.2019.00235

**Published:** 2019-09-18

**Authors:** Qinying Ma, Yuan Geng, Hua-long Wang, Bing Han, Yan-yong Wang, Xiao-li Li, Lin Wang, Ming-wei Wang

**Affiliations:** ^1^Department of Neurology, the First Hospital of Hebei Medical University, Shijiazhuang, China; ^2^Brain Aging and Cognitive Neuroscience Key Laboratory of Hebei, Shijiazhuang, China; ^3^Department of Neurology, the First Hospital of Shijiazhuang, Shijiazhuang, China; ^4^Emergency Department, CNPC Central Hospital, Langfang, China

**Keywords:** repetitive transcranial magnetic stimulation, high frequency, aged, cognitive impairment, synaptic plasticity

## Abstract

Normal aging is accompanied by hippocampus-dependent cognitive impairment, which is a risk factor of Alzheimer’s disease. This study aims to investigate the effect of high frequency-repetitive transcranial magnetic stimulation (HF-rTMS) on hippocampus-dependent learning and memory in aged mice and explore its underlying mechanisms. Forty-five male Kunming mice (15 months old) were randomly divided into three groups: aged sham, 5 Hz rTMS, and 25 Hz rTMS. Two sessions of 5 Hz or 25 Hz rTMS comprising 1,000 pulses in 10 trains were delivered once a day for 14 consecutive days. The aged sham group was treated by the reverse side of the coil. In the adult sham group, 15 male Kunming mice (3 months old) were treated the same way as the aged sham group. A Morris water maze (MWM) was conducted following the stimulation, and synaptic ultrastructure was observed through a transmission electron microscope. HF-rTMS improved spatial learning and memory impairment in the aged mice, and 5 Hz was more significant than 25 Hz. Synaptic plasticity-associated gene profiles were modified by HF-rTMS, especially neurotrophin signaling pathways and cyclic adenosine monophosphate response element binding protein (CREB) cofactors. Compared to the aged sham group, synaptic plasticity-associated proteins, i.e., synaptophysin (SYN) and postsynaptic density (PSD)-95 were increased; brain-derived neurotrophic factor (BDNF) and phosphorylated CREB (pCREB) significantly increased after the 5 Hz HF-rTMS treatment. Collectively, our results suggest that HF-rTMS ameliorated cognitive deficits in naturally aged mice. The 5 Hz rTMS treatment significantly enhanced synaptic structural plasticity and activated the BDNF/CREB pathway in the hippocampus.

## Introduction

Increased human longevity has magnified the negative impact that aging can have on cognitive integrity in older individuals (Foster et al., 2017). Normal aging is associated with declining cognitive function, which may result from changes in hippocampus circuits (Vanguilder and Freeman, 2011). Hippocampal-functional dependent learning and memory capabilities are vulnerable during brain aging (Gray and Barnes, 2015), and these involve attention, working memory, and episodic memory (Rosenzweig and Barnes, 2003).

Synaptic plasticity is crucial for the involvement of hippocampal neurocircuitry in spatial cognition (Torii et al., 2012). It is well accepted that synaptic plasticity in the normal aging process is still reserved in the hippocampus and provides opportunities for potential interventions in prodromal stages before dementia.

Repetitive transcranial magnetic stimulation (rTMS) is a safe, inexpensive, noninvasive extracranial stimulation method. Delivering electrical stimuli to excite or inhibit the brain can affect neuron activity so as to play different roles in neural modulation by adjusting stimulation parameters such as frequency and intensity (Houdayer et al., 2008). Generally, high-frequency stimulation (HF-rTMS) is considered to evoke neural excitability (Medina and Túnez, 2013). At present, rTMS is widely used in clinical treatment mainly for neurological and psychological diseases (Kim et al., 2010); for example, by ameliorating the degree of depression, reducing the motor symptoms associated with Parkinson’s disease, and regulating cognition and mood in stroke patients (Guse et al., 2010; Liao et al., 2015). Some neuroscientific studies have shown that HF-rTMS (e.g., 10 Hz, 15 Hz, or 20 Hz) applied over the left dorsolateral prefrontal cortex (DLPFC) with a motor threshold within a range of 80%–110% was likely to bring out significant cognitive improvement in adult patients or healthy volunteers (Cattaneo and Silvanto, 2008).

Since rTMS is known as state-dependent (Kim et al., 2012), whether it plays the same role in an “aged” context that it does in adults is being considered. Additionally, a five-daily 10 Hz HF-rTMS was reported to improve attentional control in normally aging individuals (Cui et al., 2013). To establish a mouse model that mimics human aging and use for general cognitive changes, an outbred stock of Kunming (KM) mice (Chen et al., 2004), derived from the Swiss albino mouse, is widely employed in studies on neuroscience. Because of the high heterogeneous background, these mice are more similar than inbred stock are to the human population. Age-related cognitive impairment in KM mice has been well confirmed (Hoogendam et al., 2010; Wang et al., 2015). Previous work has demonstrated that 25 Hz HF-rTMS improved non-spatial memory performance in aged KM mice, regulated neuronal excitability, and modified voltage-dependent Ca^2+^ channels compared to age-matched sham mice (Hoogendam et al., 2010). However, whether spatial learning and memory are influenced by HF-rTMS is to be confirmed in this study.

It has been reported that the therapeutic effects of rTMS are coordinated by several signaling pathways (e.g., brain-derived neurotrophic factor, BDNF pathway) and networks involved in the brain’s structural and functional regulation (Kim et al., 2010; Pell et al., 2011; Chang et al., 2016). rTMS-induced excitability modulation shares common characteristics with long-term potentiation (LTP)/long-term depression (LTD)-induced synaptic plasticity (Ma et al., 2014). Whether the conditioning effect of HF-rTMS on the aged brain recruits the mechanism of synaptic plasticity remains largely unclear. Ma et al. (2014) reported that the neuronal structural plasticity-related gene and protein (e.g., synaptophysin, SYN and PSD95) that were involved in low-frequency rTMS were found to reduce the improvement of cognitive function in aged mice (Vorhees and Williams, 2006). However, the mechanisms regarding synaptic plasticity for HF-rTMS in modifying age-related cognitive impairment remain to be discussed.

Different TMS frequencies have different effects on cognition in animal experiments. Zhang et al. (2015) showed that a 5 Hz frequency stimulation could improve spatial learning and memory ability in rats with vascular dementia. Other studies have shown that low-frequency magnetic stimulation can significantly improve learning and memory function in rats (Trebbastoni et al., 2016). Previous studies have also shown that low-frequency rTMS can improve learning and memory ability in aged mice; even at 1 Hz rTMS, they still showed cognitive improvement (Huang et al., 2017). However, preliminary studies from our laboratory suggest that high-frequency magnetic stimulation may be more effective than low-frequency magnetic stimulation for cognitive improvement (data not shown). Therefore, 5 Hz and 25 Hz stimulation frequencies were selected for observation in this study as the commonly used high-frequency range (5–25 Hz).

Here, we aim to identify the role and possible mechanisms of HF-rTMS on hippocampus-dependent cognitive impairment in aged mice.

## Materials and Methods

### Animals

Forty-five aged (15 months old) and 15 adult (3 months old) male KM mice were purchased from the Experimental Animal Center of Hebei Medical University (Shijiazhuang, China) and raised under temperature conditions of 20–22°C and a 12-h light-dark cycle. Mice had access to food and water *ad libitum*. All animal experiments were performed under an animal study protocol approved by the ethics committee of Hebei Medical University.

### HF-rTMS Treatment

The detailed stimulation procedures, including parameters and patterns, were described in previous research (Hoogendam et al., 2010). Briefly, HF-rTMS was performed using an MCF-B65 butterfly coil (outer diameter 90 mm) connected to a MagProX100 magnetic stimulator (active pulse width 280 μs, maximum output 4.2 T, MagVenture, Denmark). Aged mice (15 months old) were randomly divided into three groups (*n* = 15 for each group): aged sham, 5 Hz rTMS, and 25 Hz rTMS. In the rTMS groups, mice were exposed to HF-rTMS (5 Hz or 25 Hz) with the coil placed 1 cm above the mouse’s head for 14 consecutive days, 100 pulses per train, with a 30 s interval at 20% maximum output (0.84T) and 10 trains daily. In the aged sham group, mice were treated in a similar way to the aged rTMS mice using the reverse side of the coil without the rTMS effect. In the adult sham group (3 months old, *n* = 15), mice were treated in the same way as the aged sham group with the reverse side of the coil. During the procession of rTMS or sham rTMS, mice were fixed calmly with a flexible plastic tube with holes at both ends. The small hole on the head end was kept to enable them to breathe, and the bigger hole was suitable for the mice to probe into the tube; a sponge was used to fix the mice gently at the tail end.

### MWM for Spatial Learning and Memory Test

The Morris water maze (MWM) test was carried out according to the protocols modified from Morris (1984) to assess the spatial-related form of learning and memory (Han et al., 2016). The water maze was a circular pool (with a diameter of 120 cm and a height of 60 cm). The pool was divided into four quadrants (I, II, III, and IV) by two imaginary lines. The water depth in the pool was 45 cm, just 1 cm above the platform, with a temperature in the range of 20–22°C. Before the test, a non-toxic black pigment was added to make the water opaque so that the platform became invisible to the mice. The platform (8 cm in diameter) was placed in the middle of the target quadrant (e.g., quadrant I) and maintained during spatial navigation. The surroundings of the pool were shaded by curtains, and the indoor light brightness was adjusted to avoid the reflection of the water surface in the video capture system, which was 2 m above the water maze. Quiet was kept throughout training and testing. The start positions of the MWM in each trial were randomized. In the place navigation test (representing learning ability), all mice were released once at each of the four start positions on each day for five consecutive days. Mice were allowed to search for the platform for a maximum of 60 s. The time started when the rats were put into the water and ended when they found and climbed onto the platform; this was recorded as “escape latency.” Mice that could not find the platform within 60 s were led to the platform and allowed to stay for 10 s to become familiar with the environment and the platform position. In this case, escape latency was considered as 60 s. After navigation, a spatial probe trial was performed on day six. The platform was removed, and the mice were released at an additional probe position. Mice were allowed to search for the platform for 60 s. The ratio of swimming distance in the original quadrant to the total distance (distance ratio) and the time of platform crossing in the original quadrant were recorded and analyzed by the ANY-maze video-tracking analysis system (Stoelting, USA).

### Transmission Electron Microscope Analysis for Synapses

Following the procedure in our former research (Annoni et al., 2016), mice (*n* = 3/group) from the three aged groups were anesthetized and perfused with perfusion fluid containing 3% paraformaldehyde and 1% glutaraldehyde in 0.1 M PB (pH 7.4). Hippocampi were separated and stored in 4% glutaraldehyde. Hippocampi were then trimmed to approximately 1 mm^3^ and cut into sections (100 μm). The sections were fixed using osmium acid, dehydrated in ethanol, embedded in araldite, and stained with toluidine blue. The cell layer was located in the field of view of the light microscope. The sections at a thickness of 70 nm were prepared from the middle third of the CA1 stratum radiatum using an ultramicrotome and collected on Formvar-coated single-slot grids, followed by a double-stain with uranyl acetate and lead citrate. Images were captured with iTEM software for Hitachi 7500 transmission electron microscopy (Electron Microscopy Experimental Center of Hebei Medical University, Shijiazhuang, China). The average thickness of postsynaptic density (PSD), the width of synaptic cleft, and the ratio of perforated synapse and synaptic curvature were quantified and analyzed with NIS-Elements BR software.

### Real-Time Polymerase Chain Reaction Gene Array

At 7 days after rTMS, i.e., 1 day after the MWM test, mice (*n* = 6/group) in the three aged groups were sacrificed. Total RNA was isolated with the RNeasy Mini Kit (Qiagen, Valencia, CA, USA) from the hippocampus tissue. An equal amount of RNA was then converted into cDNA using SABiosciences’ RT^2^ First Strand Kit (Qiagen, Valencia, CA, USA) as per the manufacturer’s protocol. Afterward, complementary DNA was amplified by polymerase chain reaction (PCR) using RT^2^ SYBR^®^ Green qPCR Mastermix (Qiagen, Valencia, CA, USA). Synaptic plasticity gene profiling was done using a 96-well format RT^2^ Profile PCR Array Mouse Synaptic Plasticity kit (SABiosciences, Qiagen, Valencia, CA, USA) with an ABI7500 qRT-PCR instrument (Applied Biosystems, Foster City, CA, USA; Zhang et al., 2012). This array analyzed 84 genes involved in mouse synaptic plasticity, including 30 immediate-early response and two late response genes, 28 genes involved in LTP, 21 LTD genes, 19 neuronal receptors, 15 PSD genes, 10 cyclic adenosine monophosphate response element binding protein (CREB) cofactors, nine cell adhesion genes, five extracellular matrix and proteolytic processing genes as well as two other genes involved in synaptic plasticity. Data were normalized for GAPDH levels by the ΔΔCt method. The relative abundance of each mRNA species was assessed and analyzed using programs provided by the manufacturer, and individual genes were analyzed using SDS software. A gene was considered differentially regulated if the difference was ≥1.5-fold and *P*-values were <0.05 compared to the aged sham group.

### Western Blotting for Synapse Plasticity-Related Proteins

At 7 days after rTMS, the aged mice (*n* = 6/group) were sacrificed, and the hippocampus tissues were homogenized in a lysis buffer containing 20 mM Tris–HCl (pH 7.4), 0.5% Triton X-100, and Halt^TM^ protease inhibitors (87785, Thermo Scientific). Protein concentrations were determined by the Bradford method. Samples were denatured after boiling for 10 min. The proteins were separated by sodium dodecyl sulfate-polyacrylamide gel electrophoresis (SDS-PAGE) and transferred onto polyvinylidene fluoride (PVDF) membranes. The membranes were blocked in 5% skimmed milk in Tris-HCl containing 0.1% Tween-20 and then probed overnight with primary antibodies, respectively, i.e., anti-SYN (1:2,000, Abcam, Cambridge, MA, USA), anti-PSD95 (1:2,000, Abcam, Cambridge, MA, USA), anti-CREB (1:1,000, Abcam, Cambridge, MA, USA), anti-phospho-CREB (anti-pCREB; 1:2,000, Abcam, Cambridge, MA, USA), anti-brain derived neurotrophic factor (anti-BDNF, 1:2,000, Abcam, Cambridge, MA, USA), anti-β-actin (1:2,000, Abcam, Cambridge, MA, USA), and anti-GAPDH (1:2,000, Abcam, Cambridge, MA, USA). Membranes were then washed in TBST [150 mmol/L of NaCl, 50 mmol/L of Tris (pH 7.4), and 0.1% Tween-20] and incubated with anti-rabbit immunoglobulin G (IgG) horseradish-peroxidase-conjugated secondary antibodies for 2 h. The bands were immunodetected using an enhanced chemiluminescence kit (ECL, Millipore, and Temecula, CA, USA), detected using the Odyssey IR fluorescence scanning imaging system, and quantified by densitometric analysis using the ALPHA analytical system. β-actin or GAPDH was used as an internal control. The ratio of OD value was calculated for relative protein expression level (Zhang et al., 2013).

### Statistical Analysis

Data is presented as the mean ± standard error of the mean (SEM) and analyzed with SPSS 22.0 statistical software (IBM, Chicago, IL, USA). Student’s *t*-test was used for the spatial navigation test and gene differential expression. Before the *t*-test, an *F*-test was conducted to determine the equality of the variances of the two normal populations. A *P*-value < 0.05 was considered statistically significant.

## Results

### HF-rTMS Improved Spatial Learning and Memory Performance in Aged Mice

**Figure 1:**
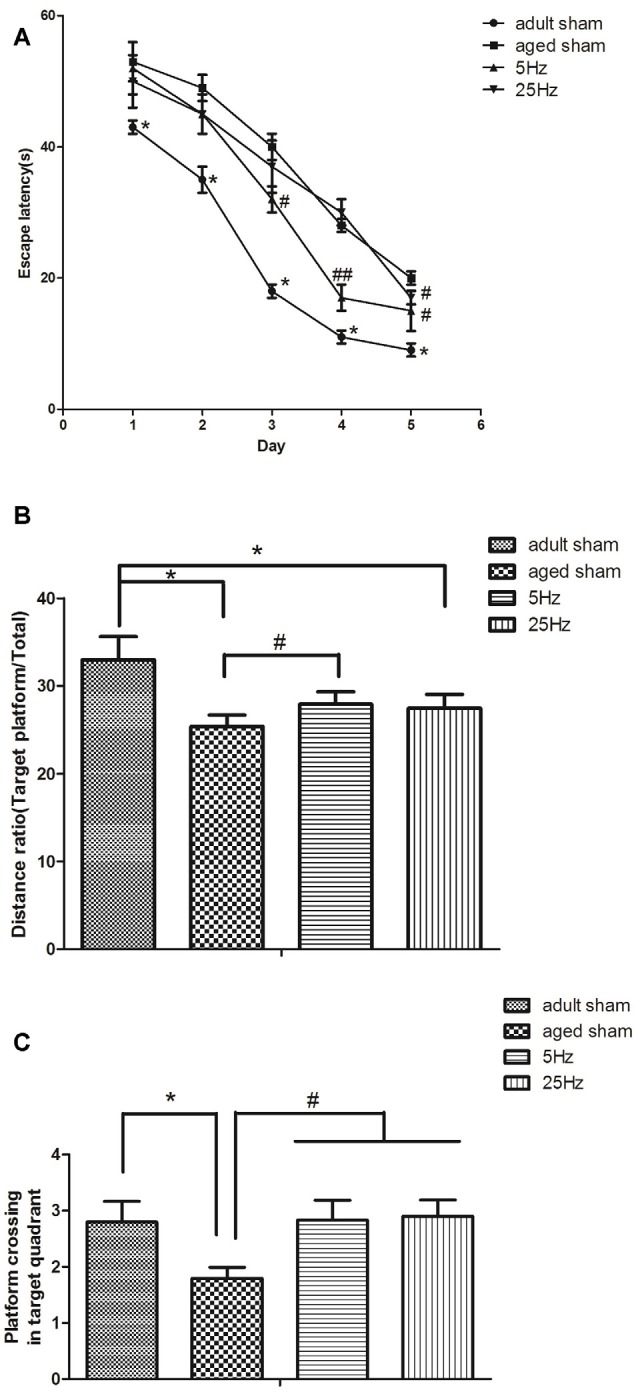
Repetitive transcranial magnetic stimulation (rTMS) is beneficial for spatial learning and memory in mice, as tested by the Morris water maze (MWM). **(A)** The mean escape latency in the spatial learning test. *P < 0.05 aged sham group vs. adult sham group; ^#^*P* < 0.05 and ^##^*P* < 0.01 compared to the aged sham group. **(B)** The ratio of swimming distance in the original quadrant to the total distance (distance ratio). *P < 0.05 vs. adult sham group; ^#^*P* < 0.05 vs. aged sham group. **(C)** The time of platform crossing in the original quadrant. *p < 0.05 vs. adult sham group; ^#^*P* < 0.05 vs. aged sham group. Data are shown as mean ± standard error of the mean (SEM); *n* = 15 for each group.

Spatial navigation: the mean escape latency in the aged groups (aged sham, 5 Hz, and 25 Hz rTMS) was significantly increased compared to the adult sham group (*P* < 0.05). Compared to the aged sham group, the mean escape latency decreased significantly in the 5 Hz (*P* < 0.01) and 25 Hz rTMS groups (*P* < 0.05, Figure 1A). Spatial probe: compared to the adult sham group, the distance ratio of the aged sham and 25 Hz groups were significantly increased (*P* < 0.05, Figure 1B), and the time of platform crossing was decreased in the aged sham group (*P* < 0.05, Figure 1C). Compared to the aged sham group, the distance ratio was significantly increased in the 5 Hz rTMS group (*P* < 0.05, Figure 1B), and the time of platform crossing was significantly increased in the 5 Hz and 25 Hz rTMS groups (*P* < 0.05, Figure 1C). In summary, spatial learning and memory were impaired in aged mice compared to adult mice. HF-rTMS improved spatial learning and memory ability in aged mice, and 5 Hz showed a more significant difference than did 25 Hz.

### Synaptic Ultrastructural Parameters Were Significantly Modulated by HF-rTMS

**Figure 2:**
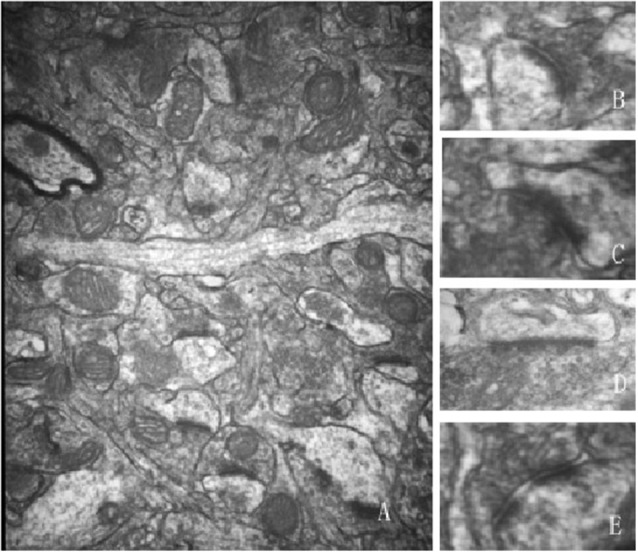
Ultrastructure alterations of the Gray I synapse were modulated and normalized by rTMS (40,000×). **(A)** A typical electron microscopic view for calculating and measuring synapses. Typical synaptic morphology is shown as: **(B)** convex shape; **(C)** concave shape; **(D)** plain shape; **(E)** perforated synapse. Synaptic parameters were calculated and analyzed after rTMS according to the microphotographs. Data are shown in Table 1.

This study focused only on excitatory synapses. PSD thickness (nm), synaptic cleft width (nm), synaptic curvature, and the number of perforated synapses were counted in typical Gray I synapses (Figure 2). Compared to the aged sham group, PSD thickness was increased, and the width of the synaptic cleft was decreased significantly in the 5 Hz rTMS group (*p* < 0.05, Table 1); no significant difference of all the parameters was found in the 25 Hz rTMS group.

**Table 1:** Comparison of synaptic parameters in aged high frequency-repetitive transcranial magnetic stimulation (HF-rTMS) groups.

	Aged sham	5 Hz rTMS	25 Hz rTMS
Number of counted synapses	78	53	52
Number and ratio of perforated synapse	18 (23.08%)	14 (26.42%)	11 (21.15%)
Thickness of PSD (nm)	48.131 ± 2.08	54.84 ± 1.25*	52.82 ± 0.90
Synaptic cleft width (nm)	15.82 ± 0.85	14.88 ± 0.94*	15.59 ± 0.54^#^
Synaptic curvature	1.0634 ± 0.0084	1.0653 ± 0.0135	1.0674 ± 0.0047

Data were shown as mean ± SEM; *P < 0.05 vs. aged sham group, ^#^P < 0.05 vs. 5 Hz rTMS group.

### Synaptic Plasticity-Associated Gene Profiles Were Modified by HF-rTMS

A different gene expression pattern was shown after a different HF-rTMS treatment. A more than 1.5-fold up-regulated or down-regulated change was considered as a differential expression (Table 2). Compared to the aged sham group, 12 genes were significantly regulated more than 1.5-fold in the 5 Hz rTMS group, and 4 of them up-regulated more than 2-fold: BDNF, AC1, PSD95, and CAMK II. The maximum up-regulated gene was BDNF at 4.22-fold. PP1 was the only significant down-regulated gene, at 2.8-fold (Table 3). As for the 25 Hz rTMS group vs. the aged sham group, three genes were found to be significantly changed, and all were down-regulated, i.e., Timp1, Tnf-α, and Nt-4/5, with a maximum of 2.64-fold for Timp1 (Table 3). CREB cofactors, IEGs, LTP, and PSD-related genes were significant based on their classification of differential gene expression. Compared with the KEGG pathway database, neurotrophin signaling pathways (in which BDNF, TrkB, Akt, CaMK, and CREB act as signaling molecules) were matched most for the 5 Hz rTMS group compared to the aged sham group; there was no significant matching for the 25 Hz rTMS group vs. the aged sham group.

**Table 2:** Total 84 synaptic plasticity associated genes were classified as 10 groups according to the functions.

Function	Gene symbol
Imediate-early response genes (IEGs)	Arc, Bdnf, Cebpb, Cebpd, Creb1, Crem, Egr1, Egr2, Egr3, Egr4, Fos, Homer1, Jun, Junb, Klf10, Mmp9 (Gelatinase B), Nfkb1, Nfkbib (Trip9), Ngf, Nptx2, Nr4a1, Ntf3, Pcdh8, Pim1, Plat (tPA), Rela, Rgs2, Rheb, Srf, Tnf
Long term potentiation (LTP)	Adcy1, Adcy8, Bdnf, Camk2a, Camk2g, Cdh2 (N-cadherin), Cnr1, Gabra5, Gnai1, Gria1, Gria2, Grin1, Grin2a, Grin2b, Grin2c, Grin2d, Mapk1, Mmp9 (Gelatinase B), Ntf5, Ntrk2, Plcg1, Ppp1ca, Ppp1cc, Ppp3ca, Prkca, Prkcc, Rab3a, Ywhaq
Long term depression (LTD)	Gnai1, Gria1, Gria2, Gria3, Gria4, Grip1, Grm1, Grm2, Igf1, Mapk1, Nos1, Ngfr, Pick1, Plat (tPA), Ppp1ca, Ppp1cc, Ppp1r14a (Cpi-17), Ppp2ca, Ppp3ca, Prkca, Prkg1
Cell adhesion	Adam10, Cdh2 (N-cadherin), Grin2a, Grin2b, Ncam1, Pcdh8, Ppp2ca, Reln, Tnf
Extracellular matrix & proteolytic processing (EMPP)	Adam10, Mmp9 (Gelatinase B), Plat (tPA), Reln, Timp1
CREB cofactors	Akt1, Camk2g, Grin1, Grin2a, Grin2b, Grin2c, Grin2d, Mapk1 (Erk2), Ppp1ca, Ppp1cc
Neuronal receptors (NR)	Ephb2, Gabra5, Gria1, Gria2, Gria3, Gria4, Grin1, Grin2a, Grin2b, Grin2c, Grin2d, Grm1, Grm2, Grm3, Grm4, Grm5, Grm7, Grm8, Ntrk2
Postsynaptic density (PSD)	Adam10, Arc, Dlg4 (Psd95), Gria1, Gria3, Gria4, Grin1, Grin2a, Grin2b, Grin2c, Grm1, Grm3, Homer1, Pick1, Synpo
Late response genes	Inhba, Synpo
Others	Kif17, Sirt1

*Underline: fold changes >1;5; P < 0.05*.

**Table 3:** Significantly changed genes for HF-rTMS vs. aged sham groups.

		Mean fold change			
Gene name	Gene symbol	5 Hz vs. Aged sham		25 Hz vs. Aged sham	
		Test sample/Control sample	*P*-value	Test sample/Control sample	*P*-value
BDNF	Bdnf	**4.22**	0.0114*	1.23	0.3471
AC1	Adcy1	**2.49**	0.0471*	1.50	0.2372
PSD-95	Dlg4	**2.43**	0.0146*	1.20	0.7400
CAMK II gamma	Camk2g	**2.10**	0.0458*	−1.02	0.8875
Akt	Akt1	1.93	0.0050*	1.50	0.0840
c-fos	Fos	1.87	0.0097*	−1.32	0.3694
GluR-A (Glur-1)	Gria1	1.78	0.0308*	−1.05	0.8485
Sir2 α	Sirt1	1.73	0.0498*	1.52	0.0841
ERK (Erk2, MAPK2, p42mapk)	Mapk1	1.70	0.0481*	−1.05	0.7515
Homer1 (PSD-Zip45)	Homer1	1.67	0.0428*	−1.05	0.7677
TrkB	Ntrk2	1.62	0.0446*	1.50	0.2324
NT-5, NT-4 (Ntf-5, Ntf4)	Ntf5	−1.44	0.7367	**−2.54**	0.0336*
TIMP-1 (Timp)	Timp1	−1.80	0.1856	**−2.64**	0.0214*
TNF-α	Tnf	−1.80	0.1856	**−2.64**	0.0214*
Ppp1c (dism2)	Ppp1ca	**−2.08**	0.0230*	−1.33	0.5346

Boldfaced character: fold changes >2. *p < 0.05.

### Effect of HF-rTMS on Synaptic Plasticity-Associated Proteins: SYN, PSD95, BDNF, and CREB

**Figure 3:**
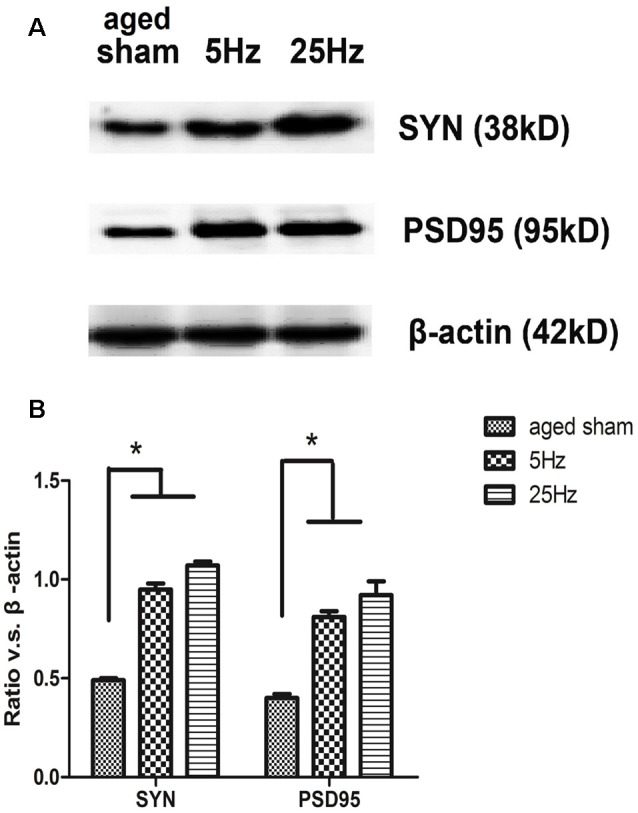
The expression of the synaptic marker in the hippocampus of aged mice after rTMS. **(A)** Western blotting bands for 7 days after rTMS. synaptophysin (SYN) and postsynaptic density (PSD)-95 levels were increased in both the 5 Hz rTMS and 25 Hz rTMS groups. **(B)** Statistic analysis of relative protein expression of SYN and PSD95. Ratio of OD value vs. β-actin is shown as mean ± SEM (*n* = 6 for each group). *p < 0.05 vs. sham group.

The pre- and post-synaptic markers assessed with western blotting are shown in Figure 3. Compared to the aged sham group, SYN and PSD95 both increased significantly in the rTMS groups. Compared to the 5 Hz rTMS group, no significant difference was found for either SYN or PSD95 in the 25 Hz group.

**Figure 4:**
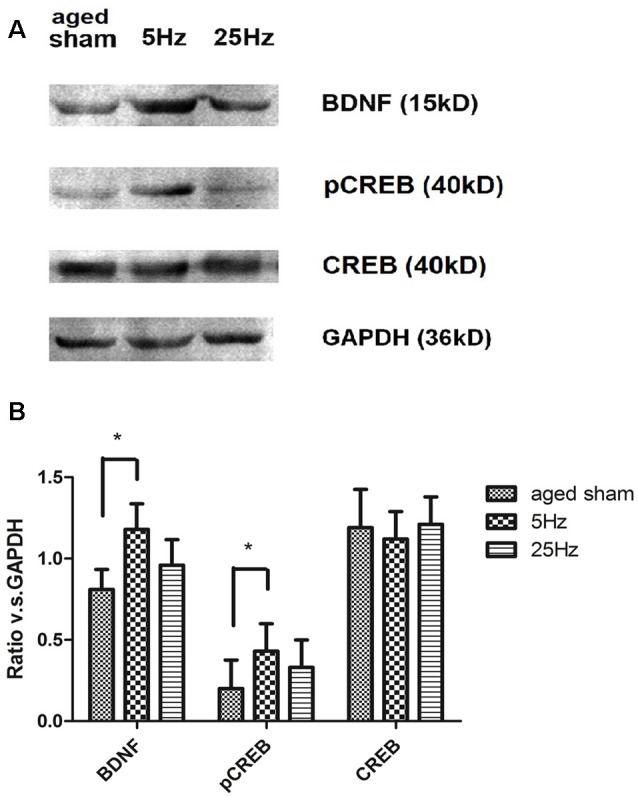
The effect of rTMS on brain-derived neurotrophic factor (BDNF), pCREB, and total CREB levels in the hippocampus 7 days after rTMS. **(A)** Western blotting bands for BDNF, total CREB, and pCREB in the hippocampus. **(B)** Statistic analysis of relative protein expression level of BDNF, total CREB, and pCREB. Ratio of OD value vs. GAPDH is shown as mean ± SEM; *n* = 6 for each group. *P < 0.05 vs. sham group.

BDNF/CREB signaling cascade-associated proteins were additionally assessed by western blotting (Figure 4). Compared to the aged sham group, BDNF and pCREB increased significantly in the 5 Hz rTMS group (*p* < 0.05) but not in the 25 Hz group. No significant difference in total CREB was found among the groups.

## Discussion

Age-related cognitive impairment is considered a pre-symptomatic phase of dementia, which may take decades to evolve (Allison et al., 2016). Aspects of spatial navigation may be particularly sensitive in detecting the earliest cognitive impairments of preclinical Alzheimer’s disease (Miniussi and Ruzzoli, 2013). Nowadays, rTMS has been demonstrated to be a key tool in the study of complex brain processes (Luber and Lisanby, 2014; Mok et al., 2016). In the present study, aged KM mice were used to study the effect of two frequencies of rTMS (5 Hz and 25 Hz) on spatial cognitive impairment *via* the MWM test. Identified with considerable evidence for the deleterious effects of aging on cognition (Zhang et al., 2015; Fabbri et al., 2016; Korthauer et al., 2016; Beason-Held et al., 2017), our behavioral data in the MWM test showed that spatial learning and memory were decreased in aged KM mice when compared to adult mice. However, coinciding with the report that 5 Hz rTMS improved the cognition of adult rats and vascular dementia (Olvera-Cortés et al., 2012; Shang et al., 2016), we found that, over 14 days, both the 5 Hz and 25 Hz HF-rTMS treatments can significantly improve spatial learning and memory ability in aged mice. Although both the 5 Hz and 25 Hz HF-rTMS stimulation groups had significant improvements in spatial learning, the extent of improvement in spatial memory appeared to be greater in the 5 Hz HF-rTMS group than in the 25 Hz HF-rTMS group. We speculated that the effects under a different frequency might delegate a different cognitive domain (Bouchard and Villeda, 2015). The 25 Hz HF-rTMS treatment might play a part in the acquisition of new information but not in memory retrieval. Despite the variability in targeted cognitive domains and outcomes, the results generally show an enhancement or uniform benefit for HF-rTMS.

At present, the effect of TMS on cognitive function is still controversial, and there are differing opinions on parameter setting, treatment duration, and efficacy evaluation methods. In recent years, most studies on the cognitive effects of TMS on healthy people tend to show that high-frequency TMS can significantly improve cognitive ability in normal subjects. Several scholars have reported that low-frequency TMS can improve the performance of working memory. Clinical experiments have found that different stimulation frequencies have different effects, sometimes even opposite effects. It was found that stimulating the left DLPFC for 2 weeks was more likely to improve cognitive function with frequencies of 5 Hz, 10 Hz, or 15 Hz, an intensity of 80%–110% motor threshold, and 10–15 consecutive sequences. Adult Patients were more likely to improve in cognitive function than were healthy volunteers. Other studies have confirmed that the cognitive improvement effect of 20 Hz TMS on Alzheimer’s patients can last for three to 6 months (Gorsler et al., 2003; Lenz et al., 2016).

Behavioral results indicated that 5 Hz and 25 Hz HR-rTMS could improve the spatial learning and non-spatial memory of aged mice. Overall, 5 Hz HF-rTMS might have a better effect on improving learning and memory than 25 Hz will.

It is becoming increasingly evident that latent plasticity, dormant in the aged CNS, could be reactivated as a means to rejuvenate cognitive functions late in life (Brehmer et al., 2014; Shen et al., 2015). Since functional plasticity has been reported in previous literature (Hoogendam et al., 2010), we focused on assessing the effects of HF-rTMS on synaptic structural plasticity. SYN and PSD95, which are markers of pre- and post-synapse, were increased by 5 Hz and 25 Hz rTMS on the protein level, suggesting a structure modification role of rTMS in the aged brain, in accordance with many studies (Vorhees and Williams, 2006; Cooperrider et al., 2014; Xiao et al., 2014; Wang et al., 2016). The increase in PSD thickness and decrease in cleft width might be the major morphological change during LTP’s induction, while the increase in the curvature of the synaptic interface and the number of perforated synapses might be responsible for its maintenance (Antonenko et al., 2016). The present result of synaptic ultrastructural parameters showed that PSD thickness increased and the cleft width decreased after HF-rTMS, especially significant in the 5 Hz group, while the synaptic curvature and percentage of perforated synapse did not significantly change. It is speculated that 5 Hz rTMS might play a role mainly in LTP induction; however, 25 Hz rTMS might not behave in the same way. The limitation of the study is that we only emphasize excitatory synapses and do not investigate inhibitory synapses on rTMS affecting GABAergic action in the hippocampus, which deserves further study.

Therefore, it is of great interest to study the putative neural mechanisms underlying synaptic plasticity changes after HF-rTMS in the particular context of the aged brain. Hippocampal pathway plasticity is associated with the ability to form novel memories in older adults (Roy et al., 2014). To investigate which plasticity-associated genes are affected by HF-rTMS, we performed an mRNA-based PCR array analysis of plasticity-related genes in the hippocampal tissues of three aged groups (Panja and Bramham, 2014; Verma et al., 2016). Results demonstrated that synaptic plasticity-associated gene profiles were modified by HF-rTMS. The genetic factor highlighted CREB cofactors (but not the CREB gene *per se*) and neurotrophin signaling pathways after the 5 Hz treatment. BDNF is a molecular neurotrophic factor that plays a key role in neuronal survival and plasticity. Decreased levels of BDNF are associated with neurodegenerative diseases with neuronal loss, such as Parkinson’s disease, Alzheimer’s disease, multiple sclerosis, and Huntington’s disease. BDNF has emerged as a regulator of stable, late-phase LTP at excitatory glutamatergic synapses in the adult brain (Ma et al., 2013; Kim et al., 2016). One of the downstream cascades activated by BDNF is the cascade linked to the transcription factor CREB pathway. Activated CREB (pCREB, the phosphorylation form of CREB) can lead to synaptic restructuring to support LTP (Paramanik and Thakur, 2013). The pCREB protein acts as a molecular switch for neuronal plasticity (Bramham and Messaoudi, 2005; Zhang et al., 2016) and is well known for its role in long-term memory. On the other hand, BDNF is also a downstream gene regulated by CREB (Carlezon et al., 2005; Ehrlich and Josselyn, 2016). Significant increases of pCREB and BDNF in the dentate gyrus of adult and aged mice were connected with better learning and spatial memory (Nam et al., 2014). BDNF acts in concert with pCREB for positive feedback regulation of structural and morphological plasticity (Hernandez and Abel, 2008).

The formation and storage of long-term memory require transcription and synthesis of new proteins (Rosa and Fahnestock, 2015). Because real-time PCR arrays hinted at noteworthy changes in BDNF and CREB cofactors in mRNA levels, which may not always correlate with protein expression (Alijanpour et al., 2015), we additionally assessed the differentially expressed genes for the BDNF, total, and phosphorylated CREB by western blotting. The pCREB/CREB ratio in the hippocampus increased in the mice that showed successful memory retrieval (Hellmann et al., 2012). In an *in vitro* system, repetitive magnetic stimulation of SH-SY5Y cells resulted in increased intracellular cAMP levels and increased pCREB. Similar results were obtained in this study that pCREB, but not CREB, was increased *in vivo* after HF-rTMS intervention. The increase in the BDNF-pCREB level may act as a modulator of synaptic plasticity, suggesting a possible mechanism for the protective role of 5 Hz HF-rTMS on memory impairment occurring during the normal aging process. Strategies that exploit upstream factors or that target specific CREB-regulated genes, rather than CREB itself, could make a promising contribution to the treatment of age-related cognition impairment. In regard to 25 Hz rTMS, mechanisms such as neurogenesis or anti-inflammation might play some role.

Because of the limitations in electrophysiological techniques for the aged brain and the limited number of aged animals, the functional change on LTP/LTD and related ion channels were not studied in this research. Furthermore, to elucidate the exact cascades of the signaling pathway involved in the process, biochemical and molecular changes will be detected with a special inhibitor and stimulator in our future work. Finally, another important consideration for future application of HF-rTMS as a new rehabilitation therapy is to investigate the intensity and coil location of HF-rTMS that improves elderly cognitive dysfunction.

Although further investigations are required to validate the results and fully understand the underlying mechanisms, in the present study, we investigated the effect of HF-rTMS on hippocampus-dependent cognitive impairment in aged mice. We reported that: (1) 5 Hz and 25 Hz HF-rTMS reverse hippocampus-dependent cognitive impairment, which (2) is accompanied by hippocampal structural plasticity. (3) These changes, especially under the 5 Hz rTMS treatment, depend at least in part on the activation of BDNF/CREB pathway.

## Ethics Statement

All animal experiments were performed under an animal study protocol approved by the ethics committee of Hebei Medical University.

## Author Contributions

The work presented here was carried out in collaboration between all authors. MW and YG defined the research theme. YG and HW carried out the laboratory experiments. BH and YW analyzed the data. XL and LW co-worked on associated data collection and interpretation.

## Conflict of Interest

The authors declare that the research was conducted in the absence of any commercial or financial relationships that could be construed as a potential conflict of interest.
